# A Cost-Effectiveness Analysis of the Sentio Bone Conduction Hearing Implant System in the Australian Healthcare Setting

**DOI:** 10.3390/jmahp14010008

**Published:** 2026-01-27

**Authors:** Magnus Värendh, Ida Haggren, Helén Lagerkvist, Maria Åberg Håkansson, Jonas Hjelmgren

**Affiliations:** 1 IHE—The Swedish Institute for Health Economics, 223 61 Lund, Sweden; ida.haggren@nek.lu.se (I.H.); jh@ihe.se (J.H.); 2 Department of Economics, Lund University, 220 07 Lund, Sweden; 3 Oticon Medical AB, 436 32 Askim, Sweden; hlag@oticonmedical.com (H.L.); mbae@oticonmedical.com (M.Å.H.)

**Keywords:** clinical effectiveness, cost-effectiveness, Sentio system, hearing loss, active transcutaneous, bone conduction hearing systems

## Abstract

Bone conduction hearing implant systems (BCHIs) are established treatments for patients with conductive or mixed hearing loss or single-sided deafness when conventional hearing aids are unsuitable. This study evaluated the cost-effectiveness of the active transcutaneous system Sentio versus a similar system, i.e., Osia in an Australian setting. Scenario analyses also compared Sentio to other systems, i.e., Ponto and Baha Attract. A Markov cohort model was adapted from a previously published source to reflect Australian practice, incorporating device acquisition, surgery, maintenance, battery replacement and adverse event management over a 15-year horizon from a healthcare perspective. Effectiveness inputs were derived from published evidence using a naïve indirect comparison. Extensive sensitivity analyses and external validation tested robustness. In the base case, Sentio was associated with lower costs and a small modelled incremental quality-adjusted life years (QALYs) gain versus Osia. Scenario analyses confirmed cost-effectiveness relative to Ponto and Baha Attract, with outcomes below the Australian willingness-to-pay threshold. Health state utility, device price and reimplantation assumptions were the most influential drivers, yet Sentio remained cost-effective in over 95% of simulations. These findings support Sentio as a clinically and economically efficient BCHI in Australia and highlight the need for direct utility and long-term durability data.

## 1. Introduction

Bone conduction hearing implant systems (BCHIs) are well-established solutions for individuals with conductive hearing loss (CHL), mixed hearing loss (MHL), or single-sided deafness (SSD), particularly when traditional air conduction hearing aids are not suitable or effective [1,2,3]. These systems bypass the outer and middle ear by transmitting sound vibrations directly to the cochlea through the skull bone, offering an alternative route to auditory perception [1].

Two primary configurations exist: percutaneous and transcutaneous systems. Percutaneous systems (e.g., Oticon Medical Ponto) have an abutment that penetrates the skin, allowing direct mechanical transmission. While effective, this design poses a risk of skin-related complications, including infections and irritation at the implant site [4,5]. To address these issues, transcutaneous systems were introduced, employing either passive or active mechanisms. Passive transcutaneous implants (e.g., Cochlear Baha Attract) use magnetic coupling through intact skin but deliver lower output due to skin attenuation. Active transcutaneous systems (i.e., Cochlear Osia, MED-EL Bonebridge and Oticon Medical Sentio) place the transducer on the bone beneath the skin, improving sound transmission and reducing skin complications while maintaining qualitative audiological outcomes [6,7,8,9].

Technological differences exist between active transcutaneous systems. The Osia system features a piezoelectric transducer and is said to deliver greater audiological gain at high frequencies [10]. The implant requires minimal bone work but has a higher profile above the bone than Sentio and Bonebridge. Sentio and Bonebridge utilise electromagnetic transducers that provide stronger low-frequency output and a smoother frequency response, potentially resulting in a more natural sound quality and improved speech understanding [9,11,12]. Despite these recent innovations, mild adverse events such as skin discomfort or inflammation can still occur, particularly with implants that have a large protrusion above the bone [13,14,15]. Nevertheless, the clinical and audiological benefits of active transcutaneous bone conduction systems are well established, supporting their role in improving hearing outcomes and quality of life [16,17].

In Australia, BCHIs are offered to patients with CHL, MHL, or SSD when conventional hearing aids are inadequate. Referral is typically via ENT (Ear, Nose and Throat) or audiology services, with device selection guided by audiometric and clinical considerations. Although the initial approach to hearing rehabilitation involves fitting air conduction hearing aids, there are patients for whom these are either unsuitable or provide inadequate sound amplification. It has been reported that one in six Australians, over 3.6 million, live with some degree of hearing loss [18]. Furthermore, approximately 3.8% of the population has moderate or severe hearing loss in their worst ear [19], making them potentially eligible for BCHIs such as Sentio, Osia, or Ponto.

From a reimbursement perspective, health systems such as Australia’s require that new implantable technologies demonstrate at least comparable clinical effectiveness to existing alternatives and that any added costs are justified by improvements in outcomes [20]. Health economic evaluations are therefore critical to inform decisions about public funding and clinical adoption of new BCHIs.

While earlier studies, such as Brunner et al. [21], have evaluated the clinical and economic performance of BCHIs (i.e., Osia and Baha Attract), no published analysis has examined the cost-effectiveness of the newly introduced active transcutaneous Sentio system. Given the need for evidence to guide reimbursement decisions and support clinical adoption in Australia, this study evaluates the cost-effectiveness of the Sentio system compared with the Osia system. To provide a broader context, a scenario analysis is also conducted, incorporating additional potential comparators such as Ponto and Baha Attract.

## 2. Materials and Methods

### 2.1. Model Structure

**Figure 1:**
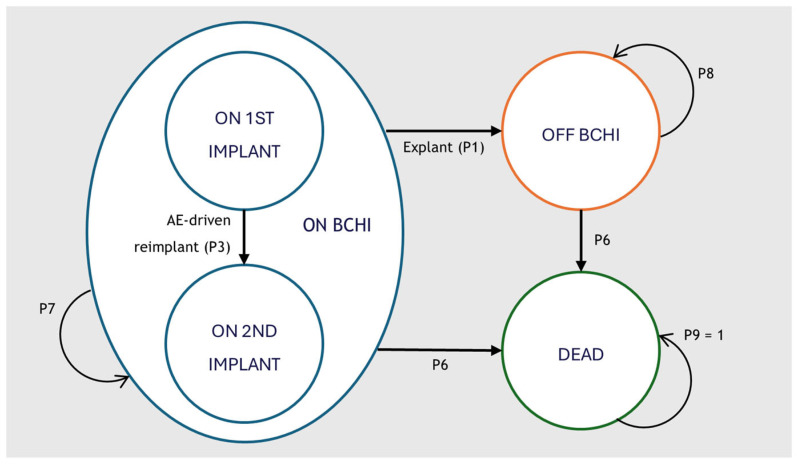
Cohort market model structure for the analysis of BCHI. The model structure is equal in all comparator arms. BCHI = Bone Conducted Hearing Implant; AE = Adverse Event.

A Markov cohort model was developed in Microsoft Excel 2016 to capture the clinical and economic pathways of patients receiving a BCHI. The model structure was adapted from the cost-effectiveness model of the Osia system developed by Brunner et al. [21] and expanded to capture health care resource use costs, cost of battery replacement and anticipated reimplantation. An overview of the structure is available in Figure 1.

The model comprised three primary health states: *on BCHI*, *off BCHI* and *dead*. All patients entered the model in the *on BCHI* health state with a first implant. Patients could transition to a second implant due to an adverse event (AE)-driven reimplantation (P3). Reimplantation was assumed to occur only once, and no patients received a third implant. Patients undergoing explantation surgery (P1) transitioned permanently to the *off BCHI* state. Mortality was applied according to age- and sex-specific population rates (P6) and was unaffected by implant status.

While in the *on BCHI* state, patients could also experience additional events that did not alter the primary health state: revision surgery (P2), anticipated reimplantation (P4) and sound processor (SP) replacement. Anticipated reimplantation represented scheduled replacement at the end of device lifespan and/or due to progressive hearing loss and could occur multiple times for eligible patients. Revision surgery reflected procedures required to manage adverse events or device malfunctions. SP replacement reflected the exchange of the external audio processor, due to wear, failure, or technological upgrades. AEs included soft tissue complications and pain. A brief description of the model events is provided in Table 1.

### 2.2. Economic Evaluation

This economic evaluation compared the Sentio system with the Osia system for patients with MHL, CHL and SSD in the Australian healthcare setting. Scenario analyses included comparisons with Ponto and Baha Attract. Model development followed the International Society for Pharmacoeconomics and Outcomes Research (ISPOR) Task Force recommendations on Good Modelling Practices [22] and adhered to the methodological requirements of Australia’s Health Technology Assessment (HTA) body, the Medical Services Advisory Committee (MSAC) [23].

### 2.3. Indirect Treatment Comparison of Clinical Effectiveness

As no direct head-to-head clinical trials were available across all relevant bone conduction hearing implant (BCHI) systems, an anchored indirect comparison was not feasible. Therefore, a naïve indirect treatment comparison (ITC) approach was implemented to estimate relative clinical effectiveness across devices. Meta-analysis was the primary source for comparison.

Effectiveness inputs were derived from published evidence for each comparator and applied directly in the model. A pooled evidence synthesis including 170 studies (>6000 implantations [24]) was used to inform follow-up–adjusted probabilities of adverse events, revisions and reimplantations for Osia, Ponto and Baha Attract. Sentio is a recently introduced device and was not included in this synthesis; instead, corresponding parameters were informed by data from its predecessor device (BCI [25]) and supplemented by available clinical trial data for Sentio [9].

Across included studies, patient populations were broadly comparable in terms of indication (CHL, MHL and SSD), baseline hearing severity and follow-up duration (Table A1 in Appendix A). The approximate distribution between CHL/MHL and SSD is five to one, and the majority of studies include adult patients only (Table A1 in Appendix A).

No formal statistical adjustment for between-study heterogeneity was performed, as the primary objective was to approximate relative effectiveness using the best available evidence. Variability in study design and definitions was explored through deterministic and probabilistic sensitivity analyses, which demonstrated the robustness of results to uncertainty in the input data.

### 2.4. Perspective, Discounting, Cycle Length and Time Horizon

The analysis was conducted from a healthcare system perspective, considering only direct medical costs; non-healthcare costs such as productivity losses or patient travel were excluded. Costs and health outcomes were discounted at an annual rate of 5% (three scenario analyses were conducted: 0% discounting for costs and QALYs; 3.5% discounting for both; and 5% for costs with 3% for QALYs), consistent with Australian health economic evaluation guidelines [23]. A half-cycle correction was applied to account for events occurring throughout the model cycle.

The model used a 6-month cycle length, with outcomes evaluated biannually. The base case time horizon was set to 15 years, which was considered sufficient to capture the lifetime costs and benefits of BCHIs while limiting long-term uncertainty. A baseline age of 40 years was selected to reflect the mean age of adults receiving BCHIs in published cohorts [14,17,25].

### 2.5. Model Inputs

#### 2.5.1. Resource Usage and Costs

The model incorporated cost categories considered to reflect the key components of implantation and long-term management of a BCHI. These included:

- BCHI acquisition
- SP replacement
- Battery replacement
- Health care resource use (routine maintenance appointments)
- Surgery-related costs for implantation, revision, reimplantation (AE-driven or anticipated) and explantation
- AE management costs

For each category, costs were estimated by multiplying unit prices by the frequency of use within each model cycle. Cycle-specific costs were then aggregated to estimate total per-patient costs over the model’s time horizon.

Healthcare resource use costs were derived from the Medicare Benefits Schedule (MBS, effective 1 January 2025) [26] and the National Hospital Cost Data Collection (NHCDC) 2020–21 public sector report [27]. NHCDC costs were adjusted for inflation using the Consumer Price Index (CPI) for Medical products, appliances and equipment from June 2021 to September 2024 [28].

Surgery and hospitalisation costs were modelled as one-time costs per procedure (implantation, revision, reimplantation, or explantation). Hospitalisation included ward, operating room and overhead costs. Anaesthesia costs were based on the MBS tariff for 61–75 min, reflecting the average duration of transcutaneous BCHI implantation. Published surgery times were 53–64 min for Osia [29,30,31] and 58 min for Sentio [9]. Ponto implantation typically does not require general anaesthesia [32], while Baha Attract was assumed to require anaesthesia in 50% of cases [33].

Following all surgeries except explantation, two audiologist appointments and one surgical follow-up were assumed. SP replacement required one audiologist appointment. For AEs (soft tissue complications and pain), two specialist appointments were included [21]. After implantation, patients attended annual audiologist appointments for SP calibration and fine-tuning. The cost of SP upgrades was incorporated at five-year intervals, consistent with Australian clinical practice. Battery cost was included, where the time to replacement was determined by battery longevity and daily use. All systems use batteries with the same cost, estimated at AUD 0.43 per battery [34,35].

All cost inputs were sourced from the latest available Australian tariffs, as of 2024, and are summarised in Table 2.

#### 2.5.2. Model Inputs for Anticipated Reimplantation and Sound Processor Replacement

Despite BCHIs typically being lifelong treatments, there is a lack of long-term clinical evidence exploring patient trajectories over a longer period, exceeding 10 years. For this reason, anticipated reimplantation and SP replacement were modelled and included as recurring events in the model to reflect device ageing, expected hearing loss progression and clinical praxis in the Australian healthcare system. Expected hearing loss progression was considered due to the relatively high proportion of patients who experience a difference in pure-tone average exceeding 10 dB over a five-year period [43]. To capture variability across patients and to avoid discrete jumps in costs and outcomes, both events were modelled using a gamma distribution.

For Osia and Baha Attract, there are no options available for stronger outputs without going through surgery. For this reason, anticipated reimplantation was assumed to occur after approximately 10 years. For Sentio and Ponto, a longer interval of 15 years was assumed, reflecting the availability of a super-powerful Sentio implant and a Ponto Superpower sound processor that would reduce the need for additional surgery. Different time horizons (5, 10, 20 and lifelong) and variations in timing of anticipated reimplantation and SP replacement, excluding the parameter of anticipated reimplantation altogether, were explored as part of the sensitivity analyses.

Across all implants, SP replacement was assumed to occur at five-year intervals, consistent with standard Australian clinical practice [44]. These values were applied as base case inputs in the model.

#### 2.5.3. Literature Search

To inform model parameters, targeted literature searches were performed in PubMed to identify evidence on utility weights, event rates and clinical effectiveness of BCHIs. While Brunner et al. [21] provided a key foundation for the Osia–Attract comparison, additional evidence was required to extend the model to include Sentio and Ponto and to identify any updated data for Osia and Baha Attract published after Brunner’s analysis.

The literature search specifically aimed to (1) include data for newer BCHI systems and comparators, (2) identify supplementary evidence on adverse event rates, device longevity and utility values, and (3) support sensitivity and scenario analyses assessing uncertainty in clinical and economic inputs.

Searches were restricted to English-language studies published from 2017 to 2024, covering Sentio, Osia (1 and 2), Baha Attract and Ponto. The strategy followed the relevant market introduction of BCHI and the approach described by Brunner et al. [21] to ensure the most recent data for comparators. Data were extracted from text and tables.

#### 2.5.4. Results of Literature Search

The targeted literature search identified several relevant studies that were used to inform and validate model inputs for clinical effectiveness, event rates and utility parameters across BCHI systems. The search identified a number of studies that could provide more recent data than Brunner et al. [21] for the comparator. A summary of the included studies is provided in Table A1 in Appendix A. Overall, the evidence base demonstrated consistent patient characteristics, follow-up durations and outcome measures across studies, supporting the assumption that BCHI populations were broadly comparable in terms of indication (CHL, MHL, SSD), baseline severity and clinical outcomes. Functional gains, patient-reported improvements and safety profiles were similar across devices, justifying the use of pooled data and naïve indirect comparisons in the model.

##### Sentio

For Sentio, evidence was limited to recent clinical studies. Reinfeldt et al. [25] reported long-term outcomes for its predecessor (BCI), with stable functional gains (~30 dB) and no serious adverse events over five years, which were used as proxy data for durability. Hol et al. [9] conducted a multicentre study of Sentio in 51 adults, reporting a mean functional gain of 32.8 dB, significant improvements in speech recognition and high patient-reported quality-of-life benefits (96%), with only mild adverse events during the first 6 months of follow-up.

##### Osia

For Osia, base case transitions were derived from the systematic review and meta-regression by Caversaccio et al. [24], synthesising data from over 170 studies (>6000 implantations). Supporting evidence from a systematic review including several multicentre trials (Key et al. [17]), showed consistent functional gains and patient-reported benefits.

##### Ponto

For Ponto, base case transitions were derived from the systematic review and meta-regression by Caversaccio et al. [24]. Additional evidence from Lagerkvist et al. [4], a systematic review of 41 studies, reported mean functional gains of 33.9 dB, high implant survival and improvements in quality-of-life outcomes (GBI mean 32.6), supporting comparability with other active BCHI systems.

##### Baha Attract

For Baha Attract, base case transitions were derived from the systematic review and meta-regression by Caversaccio et al. [24]. Supporting evidence from Kruyt et al., which showed significant audiological and PROM improvements, 100% implant survival and a low rate of soft tissue complications (~5%), was applied in scenario analyses.

#### 2.5.5. Transition Probabilities and Clinical Effectiveness Data

Transition probabilities for key events (explantation, revision surgery and AE-driven reimplantation) were sourced from systematic reviews and meta-analyses to obtain pooled, follow-up-adjusted estimates. For Osia, Ponto and Baha Attract, base case inputs were taken from Caversaccio et al. [24] to ensure consistency across comparators, while device-specific estimates from Key et al. [17] (Osia), Lagerkvist et al. [4] (Ponto) and Brunner et al. [21] (Baha Attract) were reserved for scenario analyses.

For Sentio, limited long-term data were available due to its recent introduction. Long-term outcomes from its predecessor (BCI, [25]) were therefore applied as base case inputs, assuming comparable surgical performance. Short-term Sentio data were incorporated only in scenario analyses, given the limited follow-up time of 6 months [9].

AE probabilities were derived in the same manner: pooled estimates from Caversaccio et al. [24] for Osia, Ponto and Baha Attract and Reinfeldt et al. [25] for Sentio. Minor complications in pooled sources were assumed to include both soft tissue reactions and pain, with probabilities split equally between the two categories.

For Sentio, several transition probabilities were set to zero in the base case. These represent empirical zeros based on the available evidence from Reinfeldt et al. (predecessor [25]), rather than structural assumptions of no risk. Given the limited sample size and follow-up, these zeros may reflect sparse data rather than the absence of events. To address this uncertainty, non-zero event probabilities were explored in scenario analyses by applying pooled rates for active bone conduction implants with electromagnetic transducers (aBCIem) from Caversaccio et al. [24] to Sentio.

Probabilities were assumed to be constant over the entire model time horizon. Detailed transition probabilities for key events are presented in Table 3 and AE probabilities are provided in Table 4; all estimates reflect base case inputs and are reported for a 6-month cycle length.

#### 2.5.6. Utility—Health-Related Quality of Life

Utilities for *on* and *off BCHI* health states were derived from Brunner et al. [21], who reported Health Utility Index (HUI) values for Osia and Baha Attract patients. These values were applied across Osia, Baha Attract, Ponto and Sentio to ensure comparability, in line with the approach taken for event transitions using Caversaccio et al. [24]. Subgroup adjustments (CHL/MHL and SSD) were tested in scenario analyses.

For Sentio and Ponto, the use of Osia utilities as proxies was supported by a meta-analysis of specific quality of life outcomes for percutaneous and transcutaneous bone conduction devices, showing that clinically relevant differences are more linked to the type of hearing loss than the type of BCHIs [45]. For example, the Glasgow Benefit Inventory (GBI) total score for transcutaneous was 33, 95% confidence interval [23,24,25,26,27,28,29,30,31,32,33,34,35,36,37,38,39,40,41,42,43,45] which is similar to what is reported for Sentio: 29, 95% confidence interval [9,24,25,26,27,28,29,30,31,32,33,34] and Ponto (mean 32.6 across five studies) [4,45]. Furthermore, functional gain for Osia is reported to be between 29 and 41 dB [17] and for Sentio between 30 and 36 dB [9] indicating equivalent clinical outcomes. Also, patient-reported outcomes on perceived benefits measured by the Speech Spatial and hearing Qualities (SSQ) score indicate similar outcomes for both Sentio [9] and Osia [29]. Further support was provided by clinical expert validation, who unanimously confirmed the clinical relevance of the assumption of a generalizable utility score across BCHI brands (see Section 2.7).

For Baha Attract, published utility decrements from Brunner et al. [21] were incorporated, reflecting lower quality-of-life improvements relative to active systems.

Soft tissue complication disutility was sourced from Matza et al. [46], where superficial wound infection after orthopaedic surgery was considered clinically comparable to post-implantation tissue events. Pain disutility was sourced from Dixon et al. [47] as the mean decrement for moderate pain (levels 4–7 on a 0–10 scale). This decrement was scaled to three days (3/365 of an annual decrement) to reflect its short duration.

Scenario analyses tested subgroup-specific utilities (CHL/MHL vs. SSD) using Brunner’s stratified values, while base case utilities remained common across active BCHIs. In summary, the model assumed equal utility gains across systems (Sentio, Osia, Ponto), decrements for Baha Attract and short-term disutilities for AEs. These assumptions were validated by clinical experts (see Section 2.7) and are summarised in Table 5.

### 2.6. Sensitivity Analysis

#### 2.6.1. Parameter and Scenario Analysis

A series of sensitivity and scenario analyses was conducted to explore the robustness of model outcomes.

For the one-way sensitivity analyses (SA), each parameter was varied independently within the bounds of its 95% confidence interval to assess its individual impact on incremental results. When standard errors were not available, an arbitrary standard error corresponding to ±10% of the parameter value was assumed

The scenario analyses were deterministic and focused on structural assumptions and treatment practice patterns and included the following:

- Discounting and cycle correction
  - Alternative discount rates: 3.5% and 0% [23].
  - Excluding half-cycle correction.
- Cycle length: 3-month and 12-month cycles.
- Time horizon: 5, 10, 20 and lifetime horizon.
- Patient demographics
  - Younger and older cohorts.
  - Gender-specific cohorts (100% male, 100% female).
- Device assumptions
  - Utility gains specific to CHL/MHL and SSD subgroups.
  - Alternative device acquisition costs for Sentio.
  - Alternative transition probabilities from published sources.
  - Variations in timing of anticipated reimplantation and SP replacement.
  - Alternative distributions for SP replacement and anticipated reimplantation timing (Log-Normal).
  - Exclusion of anticipated reimplantation.
- Course of care settings
  - Different numbers of annual audiologist visits.
  - Compliance variations for SP replacement and anticipated reimplantation.
  - Alternative CPI adjustments for medical costs.

#### 2.6.2. Probabilistic Sensitivity Analysis

A probabilistic sensitivity analysis (PSA) was conducted using Monte Carlo simulation with 10,000 iterations. All model parameters were varied simultaneously, with beta distributions assigned to utilities and event probabilities, and gamma distributions assigned to costs. When standard errors were not available, an arbitrary standard error corresponding to 10% of the parameter value was assumed. Parameters with zero observed events in the base case were treated as fixed in the PSA. Uncertainty related to these parameters was instead explored through dedicated scenario analyses, rather than through arbitrary continuity corrections, by assigning non-zero event probabilities derived from external pooled evidence.

### 2.7. Internal and External Validation

The model underwent internal validation through systematic quality checks to ensure technical accuracy and consistency. Programming logic was verified to confirm correct implementation of state transitions, event probabilities and discounting, while cross-checks of parameter inputs and outputs demonstrated consistency with source data. Plausibility testing of intermediate and overall results indicated that modelled outcomes followed expected patterns, supporting the reliability of the internal model structure.

For external validation, the model was benchmarked against the published framework by Brunner et al. [21], specifically replicating their Osia versus Baha Attract analysis. To ensure comparability, Brunner’s published parameters were implemented in our model. In addition, Sentio was benchmarked against Baha Attract within the Brunner framework. These steps allowed assessment of consistency with an established, peer-reviewed model. Finally, a semi-structured clinical validation process was conducted with four international experts in BCHIs, focusing on model structure, quality-of-life assumptions and anticipated reimplantation.

## 3. Results

### 3.1. Base Case Results of the Economic Evaluation

In the base case comparison, Sentio was associated with lower total costs and a small gain in QALYs, resulting in dominance over Osia. The main cost savings came from fewer anticipated reimplantations, revisions and explantations. These savings were only partly offset by higher sound processor replacement and routine healthcare use. Overall, Sentio remained both less costly and more effective than Osia. Table 6 presents the deterministic cost-effectiveness model results.

In the Australian context, this outcome falls well within the commonly accepted willingness-to-pay (WTP) threshold of AUD 50,000 per QALY [48,49], supporting Sentio’s potential inclusion on the Prostheses List and broader access for eligible patients.

### 3.2. Results of Sensitivity Analysis

#### 3.2.1. Univariate Parameter and Scenario Analysis Results

The one-way sensitivity analysis examined the impact of varying key parameters within their 95% confidence intervals on the incremental net monetary benefit of Sentio compared to Osia. The most influential drivers were the utility assigned to the *on BCHI* state, the probability of explantation for Osia and the utility of the *off BCHI* state. Other influential parameters included the assumed time to anticipated reimplantation, the initial device acquisition cost of Sentio and the interval for sound processor replacement. Across all tested ranges, Sentio remained cost-effective compared to Osia, with the incremental cost-effectiveness ratio (ICER) consistently falling within the “less costly, more effective” quadrant. Even under the most conservative assumptions, the dominance of Sentio was preserved, confirming the robustness of the base case findings.

Scenario analyses were conducted to test the model’s robustness by varying key assumptions and input parameters, one at a time, in a deterministic manner. Table A4 in Appendix B summarises the incremental percentage changes in costs, QALYs and ICERs relative to the base case.

Time horizon had the greatest impact on incremental QALYs. Reducing the time horizon from 15 to 5 years decreased QALYs by 81%, whereas extending the horizon to a lifetime increased QALYs by 215%. Changing starting age and sex had only minor effects.

Device price also influenced results. Reducing the price to AUD 13,000 lowered incremental costs by −6.9%, while increasing to AUD 15,000 raised costs by +8.5%. In both cases, Sentio maintained dominance. At a device price of AUD 23,462, the ICER reached AUD 50,000 per QALY, corresponding to the commonly assumed WTP threshold in Australia. Discounting assumptions produced similar patterns; removing discounting increased incremental QALYs by +53.5%, while applying a 3.5% discount rate reduced them by −13.1%.

Excluding anticipated reimplantation reduced cost savings by 61.8%, yet Sentio still dominated Osia. Other care-related assumptions, excluding AE pain management and audiologist appointment frequency, had a negligible impact. Two scenario analyses addressed uncertainty related to zero observed event rates for Sentio. Transition probabilities were varied using short-term Sentio data from Hol et al. [9] and pooled aBCIem event rates from Caversaccio et al. [24]. In both cases, Sentio remained dominant compared with Osia.

Overall, the scenario analyses confirmed the robustness of the base case, with Sentio remaining economically favourable compared to Osia across all tested assumptions.

#### 3.2.2. Probabilistic Sensitivity Results

**Figure 2:**
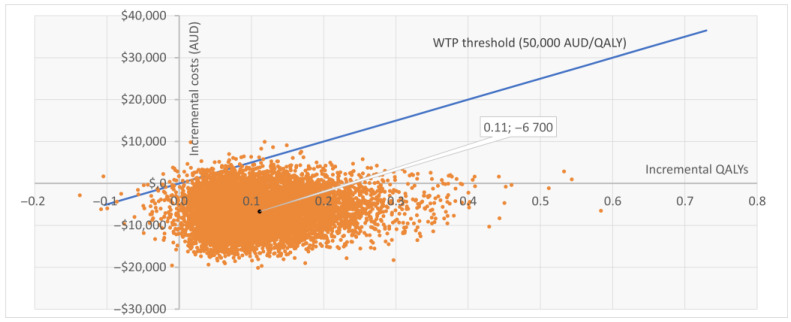
Cost-effectiveness scatter plot (CE plane) for Sentio versus Osia. Each point represents one of 10,000 PSA iterations: the majority fall below the WTP threshold line. WTP = Willingness to Pay; QALY = Quality Adjusted Life Year; AUD = Australian Dollars; PSA = Probabilistic Sensitivity Analysis.

**Figure A1:**
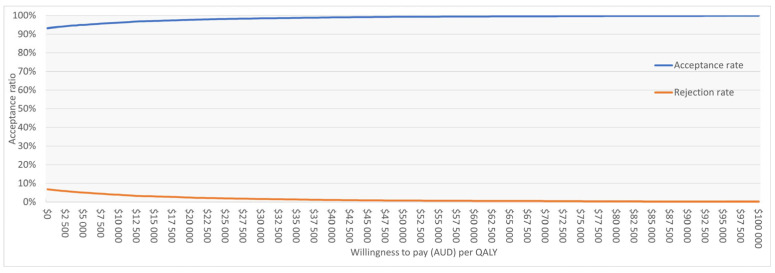
Cost-effectiveness acceptability curve (CEAC) for Sentio versus Osia. Sentio shows >95% probability of being cost-effective at willingness-to-pay thresholds below AUD 50,000 per QALY. WTP = Willingness to Pay; QALY = Quality Adjusted Life Year.

The PSA based on 10,000 iterations confirmed the robustness of the base case. Sentio incurred lower mean costs compared with Osia (AUD 39,109 vs. 45,808) and slightly higher QALYs (8.209 vs. 8.098), resulting in dominance. At a WTP threshold of AUD 50,000 per QALY, Sentio was cost-effective in 99.2% of simulations. Most iterations (92%) fell in the “less costly, more effective” quadrant, while 7% indicated “more costly, more effective,” and <1% suggested lower effectiveness. The cost-effectiveness scatter plot (Figure 2) showed nearly all iterations below the WTP threshold line. The cost-effectiveness acceptability curve (Figure A1 in Appendix B) indicated >95% probability of Sentio being cost-effective at thresholds well below AUD 50,000 per QALY, reaching near-certainty at the reference threshold.

#### 3.2.3. Results of External Validation

An external validation was conducted using the published Brunner et al. model (Osia vs. Baha Attract). Two tests were performed. First, Brunner’s published parameters were directly implemented in our model to replicate their base case. Second, Sentio was benchmarked against Baha Attract by introducing Sentio inputs into the Brunner model.

As shown in Table A5 in Appendix C, the first test produced almost identical results to Brunner’s published analysis, confirming that our model accurately replicated the original framework. In the second test, Sentio demonstrated a lower ICER than Osia when both were compared against the same benchmark (Baha Attract), reinforcing the main analysis and highlighting Sentio as the more efficient alternative.

The clinical expert validation of key model assumptions confirmed that the model structure is clinically plausible and appropriately reflects the treatment pathway, while acknowledging that it is also a necessary simplification of individual patient trajectories. Assumptions regarding utility values, anticipated reimplantation and the number of AE-driven reimplantations were judged to be reasonable and consistent with clinical practice.

### 3.3. Scenario Analyses: Sentio Compared to Ponto and Baha Attract

Scenario analyses were also conducted to assess the cost-effectiveness of the Sentio System relative to Ponto and Baha Attract (See Table A2 and Table A3 in Appendix B). The main analyses showed that, while Sentio dominated Osia in the primary comparison, the results differed when evaluated against Ponto and Baha Attract. Compared with Ponto, Sentio was associated with a positive ICER of AUD 24,200/QALY, reflecting higher incremental costs and health gains and suggesting a less favourable cost-effectiveness profile than observed in the Osia comparison. In comparison, Sentio, compared with Baha Attract, produced an ICER of AUD 11,052/QALY, indicating a slightly more favourable outcome. Both outcomes align with commonly accepted Australian WTP thresholds.

For the Sentio versus Ponto comparison, scenario analyses demonstrated that excluding anticipated reimplantation reduced the ICER (AUD 9702/QALY), highlighting the influence of long-term device replacement assumptions. In contrast, applying aBCIem-specific data from Caversaccio et al. [24] as a proxy for Sentio clinical inputs yielded minimal QALY gains (0.006 QALYs) and consequently a substantially less favourable cost-effectiveness outcome. When both adjustments were combined, the ICER increased markedly, reflecting a scenario in which small QALY differences drive disproportionate cost-effectiveness results. These relatively less favourable outcomes reflect the small incremental costs of Ponto together with the minimal incremental quality-of-life gains, which drive the ICERs to unfavourable levels under these assumptions.

For the Sentio versus Baha Attract comparison, Sentio consistently generated positive incremental QALYs with ICERs within ranges considered acceptable in Australia. Excluding anticipated reimplantation lowered the ICER further (AUD 13,632/QALY), while applying aBCIem-specific clinical inputs from Caversaccio [24] increased the ICER slightly (AUD 15,435/QALY). Combining these adjustments yielded an ICER of AUD 20,751/QALY. These results indicate that the relative cost-effectiveness of Sentio versus Baha Attract is robust, with outcomes primarily driven by assumptions on device reimplantation intervals and the size of incremental QALY gain.

## 4. Discussion

This study evaluated the cost-effectiveness of the Sentio system in the Australian setting, building on the model by Brunner et al. [21]. The findings indicate that Sentio is cost-effective compared with the Osia system, and in most scenarios even dominant, delivering a small modelled incremental QALY gain at lower costs. Scenario analyses against Ponto and Baha Attract confirmed robustness, with ICERs consistently below the Australian WTP threshold. External validation showed close concordance with Brunner’s Osia versus Attract analysis, supporting the credibility of the adapted model. Taken together, these findings strengthen the evidence base for Sentio as an efficient alternative to existing BCHIs.

The analysis has several strengths. It is based on a peer-reviewed model that was externally validated and has been expanded to incorporate additional long-term resource use and cost elements such as battery replacement and anticipated reimplantation. It is one of the first studies to systematically evaluate the cost-effectiveness of BCHIs using a structured approach to evidence collection and parameterization. External validation against Brunner’s results confirmed the accuracy of the adapted model, and the inclusion of clinical expert input further reinforced the appropriateness of the assumptions. The results are therefore highly relevant for both clinical and policy decision-making in the Australian context. A further strength of this analysis is that the model structure and key assumptions were validated by four clinical experts, who confirmed their plausibility in reflecting real-world clinical practice.

At the same time, important limitations must be acknowledged. Evidence generation in this field is constrained by the lack of head-to-head randomised controlled trials, leading to reliance on naïve comparisons. There is an inherent risk of biases of such approach stemming from disparate studies with heterogeneous designs, populations and outcome definitions. The model therefore assumes that patient populations across included studies are broadly comparable with respect to indication, baseline severity and treatment pathways. Discrepancies from population heterogeneity were mainly expected to influence clinical effectiveness and have a limited impact on safety-related parameters such as AE probabilities, reimplantation intervals and utility gains. However, Table A1 shows equivalent functional outcomes between studies, supporting the statement from clinical expertise that BCHIs is generally highly effective treatments for the targeted patient population. That is, CHL, MHL, or SSD, where traditional air conduction hearing aids are not suitable or effective.

To minimise biases from naïve comparisons and study heterogeneity, meta-analytic evidence was the first-hand choice for deriving input parameters. This reduces some of the inherited study variability and removes some of the problems with few and small individual studies. Furthermore, extensive sensitivity testing and scenario analyses were applied. Specifically, the relevant parameters were varied using wide probability distributions in a probabilistic sensitivity analysis, and alternative assumptions were explored in scenario analyses. In addition, the model assumes constant transition probabilities over time, as time-dependent event risks could not be reliably estimated due to limited long-term data. These methods ensured that uncertainty around clinical event rates was transparently reflected, allowing the robustness of the base case results to be evaluated.

A potential concern relates to the use of zero observed event rates for Sentio in the base case. These values reflect the currently available evidence rather than assumptions of no risk. To address this uncertainty, scenario analyses were conducted in which transition probabilities for Sentio were varied using both short-term Sentio data (Hol et al. [9]) and pooled aBCIem event rates from Caversaccio et al. [24]. While these scenarios substantially reduced incremental QALY gains compared with the base case, they did not alter the overall economic interpretation. This indicates that the conclusions are not driven by assumptions of zero event risk, but primarily by differences in long-term cost structure.

The analysis of patients with single-sided deafness (SSD) was limited to a single scenario and reflects a smaller patient subgroup with modest expected health gains, whereas the base case results are driven by the larger CHL/MHL population.

Health state utilities specific to Sentio were unavailable; however, reported clinical and patient-reported outcomes indicate results similar to those of Osia, as specified in Table A1 in Appendix A. Clinical expertise validation supports the assumption that the expected utilities are equal, regardless of the BCHI brand. This assumption implies that differences in device design, comfort, or cosmetic outcomes do not translate into clinically meaningful differences in generic health-related quality of life as captured by HUI. Consequently, incremental QALY differences in the model are driven by modelled event pathways and cost offsets rather than demonstrated differences in clinical effectiveness. To address the uncertainty of these assumptions, the model incorporated wide confidence intervals around the utility estimates in a probabilistic sensitivity analysis and conducted scenario analyses using subgroup-specific utilities for CHL/MHL versus SSD. Scenario analyses varying Sentio health state utilities by ±5–10% showed that Sentio remained economically favourable compared with Osia, either as a dominant option when utilities were higher or as a less costly, less effective option when utilities were lower. In addition, univariate sensitivity analysis showed that the utility weight for the *on BCHI* state was the most influential parameter, and the utility weight for the *off BCHI* state was the third most influential parameter. Overall, across all scenarios, Sentio remained economically favourable relative to Osia—either dominant or less costly and less effective, demonstrating robustness to uncertainty in utility assumptions.

Further uncertainty stems from the variability of inputs and definitions across studies and comparators, offset to some extent by the use of pooled safety evidence from Caversaccio et al. [24]. The model further assumes full compliance with sound processor replacement and anticipated reimplantation in the base case, which may not fully reflect real-world practice for all patients. Overall, these limitations contributed to uncertainty in cost-effectiveness outcomes, which was reflected in sensitivity analyses. Both the probabilistic sensitivity analysis and one-way sensitivity analysis demonstrated that incremental results varied significantly depending on assumptions, although the probability of Sentio being cost-effective remained above 95% at the Australian threshold.

Interpretation of sensitivity analyses requires caution, particularly in the context of very small incremental QALY differences. In the probabilistic sensitivity analysis, a high proportion of simulations fall within the dominance quadrant; however, this reflects the combination of modest cost differences and very small modelled QALY gains rather than evidence of consistent or clinically meaningful superiority. When incremental QALYs are close to zero, small changes in model inputs can lead to large proportional changes in ICERs, or shifts between dominance and non-dominance, despite minimal absolute differences in outcomes.

Scenario analyses further illustrate the dependence of dominance on key structural assumptions. Dominance over Osia was preserved across most scenarios; however, when assumptions related to health state utilities were altered, Sentio shifted from dominance to being less costly and less effective. This occurs because the modelled incremental QALY gain is small and largely driven by assumptions regarding utilities rather than differences in baseline clinical effectiveness. Similarly, comparisons with Ponto did not consistently result in dominance, reflecting smaller cost differentials and minimal modelled differences in QALYs. These findings highlight that dominance is not a fixed property of the intervention, but rather a function of specific modelling assumptions, particularly those related to utility inputs.

The findings raise important points for interpretation. Cost-effectiveness of Sentio was most evident in the base case, where anticipated reimplantation was included, resulting in dominance over Osia with a small modelled incremental QALY gain at lower overall costs. The magnitude of the incremental QALY gain was modest and likely within the measurement uncertainty of generic utility instruments, such as HUI, and therefore should not be interpreted as indicating clinically meaningful superiority. Differences in anticipated reimplantation rates and timing represented the largest contributor to incremental cost differences between Sentio and Osia in the model. When anticipated reimplantation was excluded, Sentio remained dominant, although the magnitude of cost savings was reduced compared with the base case. This underscores the importance of accounting for reimplantation in long-term models, since excluding it risks underestimating lifetime costs. Also, hearing loss is rarely a stable condition and a decline exceeding 10 dB in pure-tone average is relatively common over a five-year period [43] suggesting that a model should account for progressive hearing loss and associated interventions over time. Available long-term evidence furthermore suggests that reimplantation is a clinically relevant outcome [24]. This further supports its inclusion in economic analyses. However, the assumed timing of anticipated reimplantation is based on published evidence and expert opinion rather than device-specific long-term observational data and therefore represents an important modelling assumption.

Other key drivers were health-state utilities, system acquisition costs and the assumed interval to reimplantation or SP replacement, with battery replacement playing a secondary role. Pricing assumptions are particularly relevant for reimbursement considerations. At a device price of AUD 23,462, the ICER reached AUD 50,000 per QALY, corresponding to the commonly assumed WTP threshold in Australia [48,49]. This illustrates the sensitivity of cost-effectiveness to device pricing and provides a tangible benchmark for decision-makers when assessing the value of Sentio in the Australian context. These results point to clear priorities for further evidence generation, including the collection of direct utility data for Sentio using validated generic preference-based instruments such as EQ-5D or HUI, which have so far been lacking. However, these instruments may lack sensitivity to capture the full benefits of hearing implants, potentially underestimating utility gains. To strengthen future evaluations, mapping studies from device-specific patient-reported outcome measures to utility values may be more useful. The absence of Sentio-specific health state utility data increases uncertainty in the analysis, with potential impact on the ICER, and is therefore an important limitation. Such data are essential to reduce reliance on proxy measures, though alternative methods such as discrete choice experiments could provide complementary insights into WTP for specific system attributes. It should, however, be noted that the clinical expert validation revealed that there would be limited clinical relevant differences between utility scores and different brands of BCHIs. Typically, when a patient decides for treatment with BCHI, irrespective of type and brand, it is a well-grounded and motivated decision at a final step of a long journey, which usually leads to high level of satisfaction.

Future research should address the need for long-term follow-up data, particularly on reimplantation and device durability, which have a substantial impact on cost-effectiveness. Comparative evidence across CHL, MHL and SSD subgroups, as well as registry data on AEs and revisions, would improve the precision of future evaluations and reduce uncertainty.

In summary, compared with Osia, Sentio offers improved economic efficiency, and when benchmarked against Ponto and Baha Attract, it achieves favourable ICERs well below the national threshold. While the analysis necessarily relies on several structural and data-related assumptions, extensive sensitivity analyses suggest that the conclusions are robust to plausible parameter uncertainty. The results demonstrate economic favourability rather than clinical superiority and have direct implications for clinical practice and reimbursement policy, supporting Sentio as a preferred technology for patients with conductive and mixed hearing loss or single-sided deafness. The results also highlight the value of structured economic modelling in fields where randomised evidence is limited.

## 5. Conclusions

This study demonstrates that the Sentio system, assuming a baseline device price of AUD 14,000, is a cost-effective, and in many cases cost-saving, option for patients with CHL, MHL or SSD in Australia. In the base case, Sentio dominated Osia, providing higher health gains at lower overall costs. Scenario analyses against Ponto and Baha Attract confirmed robustness, with ICERs consistently below the commonly assumed national WTP threshold. Sensitivity analyses showed that results were resilient to uncertainty in key parameters, underscoring the reliability of the findings. These results support Sentio as a clinically and economically efficient alternative within the Australian healthcare setting and provide a benchmark for reimbursement and policy decisions. Future research should prioritise the collection of direct utility data and long-term evidence on reimplantation and device durability to reduce current uncertainties.

## Figures and Tables

**Table 1:** Description of model events.

Event	Explanation
Explantation (P1)	The proportion of patients transitioning from *on BCHI* to *off BCHI* due to explantation surgery.
Revision surgery (P2)	The proportion of patients undergoing the event revision surgery. Occurs only while *on BCHI* and does not transition the patient to another health state.
AE-driven reimplantation (P3)	The proportion of patients transitioning from on 1st implant to on 2nd implant due to AE-driven reimplantation.
Anticipated reimplantation (P4)	The proportion of patients undergoing the event anticipated reimplantation. Only occurs while *on BCHI* and does not transition the patient to another health state.
SP replacement (P5)	The proportion of patients changing sound processors. Only occurs while *on BCHI* and does not transition the patient to another health state.
Mortality (P6)	The proportion of patients dying in each model cycle. Mortality is driven by general population mortality, as none of the implants influence mortality. Transitions patients from *on BCHI* or *off BCHI* to *dead*.
Stay in *On BCHI* health state (P7)	The proportion of patients remaining in the health state in each model cycle.
Stay in *Off BHCI* health state (P8)	The proportion of patients remaining in the health state in each model cycle.
*Dead* (P9)	*Dead* is an absorbing health state. All patients will stay in the health state.

BCHI = bone conduction hearing implant; SP = Sound processor; AE = adverse event.

**Table 2:** Healthcare costs and sources for economic evaluation.

Cost Item	Cost (AUD)	Source
BCHI system—Sentio system	14,000.00	Baseline price within market range
BCHI system—Osia system	14,369.00	PLB; billing code QQ642 [36]
BCHI system—Ponto system	9030.00	PLB; billing codes O1039 + O1026 [36]
BCHI system—Baha Attract system	8571.00	PLB, billing code CO051 + CO068 + CO087 [36]
Replacement SP—Sentio system	6808.00	Baseline price within market range
Replacement SP—Osia system	6808.00	PLB; billing code QQ722 [36]
Replacement SP—Ponto system	6484.00	PLB; billing code O1039 [36]
Replacement SP—Baha Attract system	6484.00	PLB; billing code CO087 [36]
Surgery—Implantation	680.30	MBS; Item 41603 (Implantation of BCHI device) [26]
Surgery—Anaesthesia	225.50	MBS; Items 21120 (Initiation of anaesthesia) + 23055 (Anaesthesia 61–75 min) [26]
Hospitalisation	5588.26	IHACPA; Item D12B (Hospital cost for minor ear intervention item), total cost minus prosthesis cost) [27]. Inflated to Sep 2024 prices [28]
Post-surgery audiologist visits	350.80	MBS; Item 82301 (2× audiologist visit for implant and SP programming) [26].
Post-surgery specialist visit	49.75	MBS; Item 105 [26]
SP replacement audiologist visit	175.40	MBS; Item 82301 [26]
AEs (soft tissue complications and pain)	148.70	MBS; Items 104 (initial specialist visit) + 105 (subsequent specialist visit) [26]
Health care resource use	87.70	MBS; Item 82301 (audiologist implant/SP programming) [26]
Battery—Sentio system	11.82	Cost per 6-month cycle [9,37]
Battery—Osia system	28.64	Cost per 6-month cycle [38]
Battery—Ponto system	11.29	Cost per 6-month cycle [39,40]
Battery—Baha Attract system	5.10	Cost per 6-month cycle [41,42]

PLB = Prosthesis List Benefit; MBS = Medicare Benefits Schedule; IHACPA = Independent Health and Aged Care Pricing Authority; BCHI = bone conduction hearing implant; AE = adverse event; AUD = Australian dollars; SP = sound processor.

**Table 3:** Transition probabilities for main events (explantation, revision surgery and AE-related reimplantation) applied in the base case analysis, reported for a 6-month cycle length.

Comparator	Event	Transition Probability	SE	Source
Sentio	Explantation	0	0	Reinfeldt et al. [25]
	Revision surgery	0	0	
	AE-driven reimplantation	0.0044	0.0044	
Osia	Explantation	0.0085	0.0039	Caversaccio et al. [24]
	Revision surgery	0.0150	0.0103	
	AE-driven reimplantation	0.0055	0.0027	
Ponto	Explantation	0.0075	0.0013	Caversaccio et al. [24]
	Revision surgery	0.0468	0.0066	
	AE-driven reimplantation	0.0040	0.0015	
Baha Attract	Explantation	0.0125	0.0033	Caversaccio et al. [24]
	Revision surgery	0.0080	0.0037	
	AE-driven reimplantation	0.0040	0.0015	

AE = Adverse event; SE = Standard Error.

**Table 4:** Adverse event probabilities (soft tissue complications and pain) applied in the base case analysis, reported for a 6-month cycle length.

Comparator	Event	Transition Probability	SE	Source
Sentio	Soft tissue complication	0	0	Reinfeldt et al. [25]
	Pain	0.0088	0.0060	
Osia	Soft tissue complication	0.0638	0.0240	Caversaccio et al. [24]
	Pain	0.0638	0.0240	
Ponto	Soft tissue complication	0.0855	0.0092	Caversaccio et al. [24]
	Pain	0.0855	0.0092	
Baha Attract	Soft tissue complication	0.0833	0.0170	Caversaccio et al. [24]
	Pain	0.0833	0.0170	

SE = Standard Error.

**Table 5:** Utility weights for economic evaluation.

Health State/Event		Utility	SE	Source
All patients	*On BCHI*	0.76	0.038	Brunner et al. [21]
	*Off BCHI*	0.67	0.028	Brunner et al. [21]
CHL/MHL patients	*On BCHI*	0.73	0.040	Brunner et al. [21]
	*Off BCHI*	0.62	0.031	Brunner et al. [21]
SSD patients	*On BCHI*	0.86	0.097	Brunner et al. [21]
	*Off BCHI*	0.84	0.070	Brunner et al. [21]
Utility decrements for Baha Attract (On treatment)	All patients	−0.03	-	Brunner et al. [21]
	CHL/MHL patients	−0.06	-	Brunner et al. [21]
	SSD patients	−0.01	-	Brunner et al. [21]
AE decrements (per AE)	Soft tissue complications	−0.030	0.006	Matza et al. [46]
	Pain	−0.002	0.001	Dixon et al. [47]

Footnote: Utility weights were implemented in the economic model to derive QALYs associated with each health state. Utility decrements for the Baha Attract system were applied to reflect lower health-related quality of life relative to active transcutaneous systems. Short-term disutilities for adverse events were incorporated on a per-event basis to capture time-limited reductions in utility. SE = standard error; CHL = conductive hearing loss; MHL = mixed hearing loss; SSD = single-sided deafness; AE = adverse event.

**Table 6:** Deterministic cost-effectiveness model results, Sentio vs. Osia, discounted.

Deterministic Results	Sentio System	Osia System	Difference
Disaggregated Costs (AUD)			
Implantation	$21,068	$21,437	−$369
Revision	$0	$1911	−$1911
Anticipated reimplantation	$3152	$7505	−$4353
AE-driven reimplantation	$1206	$1385	−$179
Explantation	$0	$1028	−$1.028
SP replacement	$10,689	$9432	$1.257
HCRU	$1871	$1697	$174
Battery replacement	$252	$554	−$302
AE	$28	$367	−$339
Total costs (AUD)	$38,267	$45,316	−$7050
Total QALYs	8.205	8.094	0.112
Total Life Years	10.797	10.797	0.000
ICER			Dominating

AUD = Australian Dollars; AE = Adverse Events; SP = Sound Processor; HCRU = Health Care Resource Use; QALY = Quality Adjusted Life-Years; ICER = Incremental Cost-Effectiveness Ratio.

## Data Availability

All data were extracted from published sources. Formulas extracted from public sources will be available on request, directed to M.V.

## Abbreviations

The following abbreviations are used in this manuscript:

aBCIem	Active bone conduction implant (electromagnetic)
AE	Adverse Event
AUD	Australian Dollar
BAHS	Bone Anchored Hearing System
BCHI/BCHIs	Bone Conduction Hearing Implant System
BCI	Bone Conduction Implant, predecessor to Sentio
CEAC	Cost-Effectiveness Acceptability Curve
CHL	Conductive Hearing Loss
CPI	Consumer Price Index
GBI	Glasgow Benefit Inventory
HCRU	Health Care Resource Use
HTA	Health Technology Assessment
HUI	Health Utilities Index
HUI3	Health Utilities Index Mark 3
ICER	Incremental Cost-Effectiveness Ratio
IHACPA	Independent Health and Aged Care Pricing Authority
ISPOR	International Society for Pharmacoeconomics and Outcomes Research
ITC	Indirect Treatment Comparison
MBS	Medicare Benefits Schedule
MHL	Mixed Hearing Loss
MSAC	Medical Services Advisory Committee
NHCDC	National Hospital Cost Data Collection
pBAHS	Percutaneous bone anchored hearing systems
PLB	Prosthesis List Benefit
PROMs	Patient-Reported Outcome Measures
PSA	Probabilistic Sensitivity Analysis
PTA4	Pure-tone average (0.5, 1, 2 and 4 kHz)
QALY	Quality-Adjusted Life Year
SA	Sensitivity Analysis
SNR	Signal-to-Noise Ratio
SP	Sound Processor
SPL	Sound Pressure Level
SRS	Speech Recognition Score
SRT	Speech Reception Threshold
SSD	Single-Sided Deafness
SSQ	Speech Spatial and hearing Qualities
WTP	Willingness-to-Pay

## Appendix A. Studies Included in the Literature Search

**Table A1:** Evidence table of BCHI Studies.

Refence	Design	Device	N/Studies Cohort	Follow-Up	Functional Gain	PROMs	Safety
Key et al. (2023) [17]	Systematic review and meta-analysis	Osia	14 studies, 15 cohorts (*n* = 314) 80% CHL/MHL 20% SSD All ages	Varied	35 dB SPL 95% CI [29; 41], *n* = 140 (37.7 dB SPL CHL/MHL, *n* = 97)	Comparable or improved	Moderate to severe 3% (*n* = 222)
Lagerkvist et al. (2020) [4]	Systematic review	Ponto	41 studies (*n* = 1352) 85% CHL/MHL 15% SSD All ages	Up to 5 years	~33.9 dB, *n* = 89	98% improved GBI score, *n* = 176	Implant loss 2%, *n* = 1166 Skin reactions 15%, *n* = 863
Kruyt et al. (2020) [50]	Prospective multicentre study	Baha Attract	*n* = 54 72% CHL/MHL 28% SSD Adults	2 years	FG not mentioned, significant audiological improvement	Improved HUI3, APHAB, SSQ	Explantation 7%, skin reactions 5%, substantial pain 6%
Reinfeldt et al. (2022) [25]	Prospective multicentre study	BCI	*n* = 16 100% CHL/MHL Adults	Up to 5 years	29.5 dB (std: 7.2 dB)	Comparable	No serious AEs
Hol et al. (2025) [9]	Prospective multicentre study	Sentio	*n* = 51 76% CHL/MHL 24% SSD Adults	6 months	33 dB 95% CI [30; 36] PTA4, *n* = 51, (CHL, MHL, SSD)	96% improved GBI score	No serious adverse events
Caversaccio et al. (2025) [24]	Systematic review and meta-regression	All BCHI	170 studies (*n* = 6215; 6451 implantations) CHL/MHL/SSD All ages	Adjusted for FU time	Not applicable (safety study)	Not reported	Lowest AE rates in aBCIem (9.1/100 pt-yrs), highest major/revisions in pBAHS
Brunner et al. (2024) [21]	ITC and cost–utility model	Osia vs. Baha Attract	Osia *n* = 80, Baha Attract *n* = 54 76% CHL/MHL 24% SSD Adults	6–24 months	FG not reported, PTA4 + 7.05 dB; quiet + 15.35%; noise + 9.44 dB	HUI3: Osia 0.12, Attract: 0.06	Osia 1 explantation, Baha Attract 4 explantations, soft tissue AE lower with Osia than Attract

AE = adverse event; aBCIem = active bone conduction implant (electromagnetic); BCHI = bone conduction hearing implant system; CHL = conductive hearing loss; CI = confidence interval; FG = functional gain; FU = follow-up; HUI3 = Health Utilities Index Mark 3; MHL = mixed hearing loss; PROMs = patient-reported outcome measures; pBAHS = percutaneous bone anchored hearing system; PTA4 = pure-tone average (0.5, 1, 2 and 4 kHz); SPL = sound pressure level; SSD = single-sided deafness.

## Appendix B. Results from the Scenario Analysis

**Table A2:** Deterministic cost-effectiveness model results, Sentio vs. Ponto, discounted.

Deterministic Results	Sentio System	Ponto System	Incremental Change
Disaggregated Costs (AUD)			
Implantation	21,068	15,160	5909
Revision	0	5840	−5840
Anticipated reimplantation	3152	1571	1581
AE-driven reimplantation	1206	609	598
Explantation	0	886	−886
SP replacement	10,689	9127	1562
HCRU	1871	1716	155
Battery replacement	252	221	31
AE	28	498	−470
Total costs (AUD)	$38,267	$35,627	$2639
Total QALYs	8.205	8.096	0.109
Total Life Years	10.797	10.797	0.000
ICER			24,200

AUD = Australian Dollars; AE = Adverse Events; SP = Sound Processor; HCRU = Health Care Resource Use; QALY = Quality Adjusted Life-Years; ICER = Incremental Cost-Effectiveness Ratio.

**Table A3:** Deterministic cost-effectiveness model results, Sentio vs. Baha Attract.

Deterministic Results	Sentio System	Baha Attract System	Incremental Change
Disaggregated Costs (AUD)			
Implantation	21,068	15,639	5429
Revision	0	976	−976
Anticipated reimplantation	3152	4333	−1180
AE-driven reimplantation	1206	609	597
Explantation	0	1447	−1447
SP replacement	10,689	8480	2209
HCRU	1871	1622	249
Battery replacement	252	94	158
AE	28	458	−430
Total costs (AUD)	$38,267	$33,660	$4607
Total QALYs	8.205	7.769	0.436
Total Life Years	10.797	10.797	0.000
ICER			10,558

AUD = Australian Dollars; AE = Adverse Events; SP = Sound Processor; HCRU = Health Care Resource Use; QALY = Quality Adjusted Life-Years; ICER = Incremental Cost-Effectiveness Ratio.

**Table A4:** Scenario analysis results for Sentio versus Osia.

Setting	Scenario	Incremental Cost (% Change from Base Case)	Incremental QALY (% Change from Base Case)	ICER (AUD/QALY)
Core settings				
Population	MHL/CHL	+0.0%	+18.0%	Dominant
Population	SSD	+0.0%	−59.7%	Dominant
Health state transition probability source for Sentio	Hol et al. [9]	+12.9%	−3.9%	Dominant
Health state transition probability source for Sentio	Caversaccio et al. [24]	−4.7%	−92.0%	Dominant
Time horizon	5 years	−73.3%	−81.0%	Dominant
Time horizon	10 years	−16.5%	−44.3%	Dominant
Time horizon	20 years	−29.0%	+45.3%	Dominant
Time horizon	Lifetime (70 years)	−20.8%	+215.3%	Dominant
Starting age	70 years	−7.8%	−16.4%	Dominant
Starting age	20 years	+0.4%	+1.0%	Dominant
Percent male	0%	+0.2%	+0.3%	Dominant
Percent male	100%	−0.2%	−0.3%	Dominant
Price of Sentio system	AUD 13,000	+18.6%	+0.0%	Dominant
Price of Sentio system	AUD 14,369	−6.9%	+0.0%	Dominant
Price of Sentio system	AUD 15,000	−18.6%	+0.0%	Dominant
Utility values for Sentio compared to Osia	+5%	+0.0%	+368.1%	Dominant
Utility values for Sentio compared to Osia	+10%	+0.0%	+735.8%	Dominant
Utility values for Sentio compared to Osia	−5%	+0.0%	−368.1%	Less costly, Less effective (23,579 AUD saved for each QALY lost)
Utility values for Sentio compared to Osia	−10%	+0.0%	−735.8%	Less costly, Less effective (9944 AUD saved for each QALY lost)
Technical settings				
Distribution—SP replacement and anticipated reimplantation	Log-normal	−0.6%	+0.0%	Dominant
Half-cycle correction	No	+1.1%	+0.0%	Dominant
Cycle length	3 months	−4.3%	−4.1%	Dominant
Cycle length	12 months	+8.0%	+8.7%	Dominant
Discount rate (costs, QALYs)	0% (undiscounted)	+32.4%	+53.5%	Dominant
Discount rate (costs, QALYs)	3.5%	+8.8%	+13.1%	Dominant
Discount rate (costs, QALYs)	5%; 3%	+0.0%	+17.9%	Dominant
CPI data	General CPI	+7.3%	+0.0%	Dominant
Course of care settings				
Include anticipated reimplantation	Exclude	−61.8%	+0.0%	Dominant
Anticipated reimplantation same for both comparators	15 years	−69.5%	+0.0%	Dominant
Anticipated reimplantation same for both comparators	10 years	−76.3%	+0.0%	Dominant
Include AE pain	Exclude	−2.2%	−2.0%	Dominant
Number of annual audiologist appointments	0.8	+0.5%	+0.0%	Dominant
Number of annual audiologist appointments	1.2	−0.5%	+0.0%	Dominant
SP replacement compliance rate	0.8	+3.6%	+0.0%	Dominant
Anticipated reimplantation rate	0.8	−12.4%	+0.0%	Dominant

AUD = Australian Dollar; QALY = Quality Adjusted Life Year; ICER = Incremental Cost-Effectiveness Ratio; CHL = Conductive hearing loss; MHL = Mixed hearing loss; SSD = Single-sided deafness; AE = Adverse Event; SP = Sound Processor; CPI = Consumer Price Index.

## Appendix C. Results from External Validation

**Table A5:** External validation results comparing outcomes from the Brunner et al. [21] model with those generated by the current analysis.

External Validation Test	ICER (Model in This Article)	ICER (Model in Brunner et al. [21])	ICER Deviation	Incremental Costs Change	Incremental QALYs Change
Test 1: Brunner parameters	29,268 AUD/QALY	29,301 AUD/QALY	−0.1%	+12%	+10%
Test 2: Sentio vs. Baha Attract (structural alignment)	25,736 AUD/QALY	29,301 AUD/QALY	−12%	+1%	+13%

ICER = incremental cost-effectiveness ratio; QALY = quality-adjusted life year; AUD = Australian Dollars.
